# Multimodal neuroimaging investigation of post-stroke fatigue in middle-aged and older adults: combining resting-state fMRI and DTI-ALPS analysis

**DOI:** 10.3389/fnagi.2025.1583655

**Published:** 2025-05-13

**Authors:** Jingjing Sun, Yanlin Tang, Wenni Wang, Yong Zhang, Hengshan Ji, Peng Wang

**Affiliations:** ^1^Department of Nuclear Medicine, Jinling Hospital, Medical School of Nanjing University, Nanjing, China; ^2^School of Medical Imaging, Bengbu Medical University, Bengbu, China; ^3^Department of Cardiology, Jinling Hospital, Medical School of Nanjing University, Nanjing, China; ^4^MR Research, GE Healthcare, Shanghai, China; ^5^The First Affiliated Hospital of USTC, Division of Life Sciences and Medicine, University of Science and Technology of China, Hefei, Anhui, China

**Keywords:** post-stroke fatigue, multimodal imaging, RS-fMRI, DTI, middle-aged and older adults

## Abstract

**Background:**

Post-stroke fatigue (PSF) is a prevalent but often overlooked complication, particularly in middle-aged and older stroke survivors. Neuroimaging studies exploring the neural mechanisms of PSF in this age group are limited. This study aimed to identify imaging markers for PSF in middle-aged and older adults using a multimodal imaging approach.

**Methods:**

This retrospective case–control study analyzed data from patients with first ischemic stroke aged 50 years and above who were treated from January 2021 to June 2022 at the First Hospital of the University of Science and Technology of China. PSF was assessed using the Fatigue Severity Scale (FSS) and diagnostic criteria. All patients underwent resting-state functional MRI (rs-fMRI) and diffusion tensor imaging along perivascular space (DTI-ALPS) to assess brain functional connectivity and glymphatic system function.

**Results:**

The PSF group (mean age 62.7 ± 10.50 years) showed reduced global functional connectivity compared to non-fatigued controls (mean age 58.40 ± 9.20 years) (*p* < 0.05, FDR corrected), with enhanced connectivity between the insula and right inferior frontal gyrus. DTI-ALPS analysis revealed a negative correlation between DTI-ALPS index and fatigue severity (R^2^ = 0.40, *p* < 0.001) in the PSF group, suggesting an association between impaired glymphatic clearance and fatigue symptoms in middle-aged and older stroke survivors.

**Conclusion:**

This multimodal neuroimaging study highlights altered brain functional connectivity and glymphatic dysfunction as potential neural correlates of PSF in middle-aged and older adults. The findings provide novel insights into the complex pathophysiology of PSF in the aging brain, implicating both functional brain networks and the glymphatic system. Further research is warranted to validate these age-specific findings and explore targeted interventions for PSF in older stroke survivors.

## Introduction

Post-stroke fatigue (PSF) represents one of the most challenging sequelae affecting stroke survivors, with a reported prevalence ranging from 35 to 92% (Owens et al., 2009). PSF is characterized by a persistent and debilitating state of physical and mental exhaustion that occurs independently of physical exertion and shows minimal improvement with rest (Wu et al., 2015). This chronic fatigue significantly impairs patients’ rehabilitation outcomes, daily functioning, and quality of life (Paudel et al., 2023), while also being associated with increased mortality and disability rates (Duncan et al., 2012).

Despite its high prevalence and substantial impact, the underlying mechanisms of PSF remain poorly understood. Current diagnostic approaches rely heavily on subjective measures, primarily the Fatigue Severity Scale (FSS) (Ozyemisci-Taskiran et al., 2019). However, this assessment method presents significant limitations: the frequent co-occurrence of PSF with depression and sleep disorders complicates accurate diagnosis, and there is a critical lack of objective biomarkers to guide clinical management (Markus, 2023).

Recent advances in neuroimaging have opened new avenues for understanding the pathophysiology of PSF. Resting-state functional magnetic resonance imaging (rs-fMRI) studies have revealed alterations in multiple brain networks, particularly the default mode network and salience network, in PSF patients (Ren et al., 2024). Additionally, evidence suggests that white matter integrity may play a crucial role in PSF development (Tang et al., 2014). However, most investigations have been limited to single imaging modalities, leaving the complex interplay between structural and functional brain changes largely unexplored.

Emerging evidence highlights the crucial role of the glymphatic system (brain’s lymphatic system) in maintaining neurological homeostasis through metabolic waste clearance (Jessen et al., 2015). The recent development of diffusion tensor imaging along perivascular space (DTI-ALPS) offers a novel, non-invasive approach to assess glymphatic system function. Given that PSF may be linked to impaired metabolic clearance, a multimodal imaging approach could provide unprecedented insights into its underlying mechanisms.

This study aims to comprehensively investigate the neural correlates of PSF by combining rs-fMRI, DTI, and DTI-ALPS techniques to examine brain functional connectivity, white matter integrity, and glymphatic system function. We hypothesize that: (1) PSF patients exhibit distinct patterns of altered functional connectivity; (2) compromised white matter integrity contributes to PSF development; and (3) glymphatic system dysfunction represents a key pathophysiological mechanism in PSF. This combined multimodal imaging approach may identify novel biomarkers for PSF and inform more effective therapeutic strategies.

## Materials and methods

### Participants

This study employed a retrospective case–control design. We analyzed clinical and imaging data from patients with first-ever ischemic stroke who were treated at our institution between January 2021 and June 2022. Based on established fatigue criteria using the Fatigue Severity Scale, participants were categorized into PSF (case) and non-PSF (control) groups. All neuroimaging and clinical assessments were conducted as part of routine clinical care within 3 days of hospital admission, and data were retrospectively collected for analysis. The inclusion criteria were as follows: (1) age between 50 and 80 years; (2) first-ever ischemic stroke confirmed by magnetic resonance imaging (MRI) or computed tomography (CT); (3) within 2 weeks of stroke onset; (4) able to undergo MRI scans; and (5) willing to provide written informed consent. The exclusion criteria were: (1) severe aphasia, neglect, or cognitive impairment that precluded completion of the questionnaires; (2) pre-stroke fatigue or other neurological or psychiatric disorders that could cause fatigue; (3) severe medical conditions that could cause fatigue, such as anemia, hypothyroidism, or infection; and (4) use of medications that could cause fatigue, such as sedatives or sleeping pills. Moreover, all patients underwent the Mini-Mental State Examination (MMSE) to assess their cognitive function. Patients with an MMSE score ≤ 10 or those with an MMSE score between 11 and 23 who were considered to have poor cognitive ability were excluded from the study (Braley et al., 2014).

The study was approved by the local ethics committee (Medical Research Ethics Committee, The First Affiliated Hospital, University of Science and Technology of China, 2025KY No. 63). The Ethics Committee agreed to grant an amnesty for informed consent, which does not require the patient to sign an informed consent form.

### Clinical assessment

Information on demographics, past medical history and vascular risk factors was obtained during the interview. Body mass index (BMI) was defined as weight (kg) divided by height (m) squared (Criqui et al., 1982). Stroke etiology was classified according to the Trial of Org 10,172 in Acute Stroke Treatment (TOAST) criteria, which categorizes ischemic strokes into five subtypes: (1) large-artery atherosclerosis, (2) small-vessel occlusion, (3) cardioembolism, (4) stroke of other determined etiology, and (5) stroke of undetermined etiology. The TOAST classification is based on clinical features, neuroimaging findings, and ancillary diagnostic tests, and has been widely used in stroke research to better understand the heterogeneity of ischemic stroke and its impact on outcomes.

Other clinical assessments included the National Institutes of Health Stroke Scale (NIHSS) for stroke severity, modified Rankin Scale (mRS) for functional disability, and Mini-Mental State Examination (MMSE) for cognitive function. The NIHSS is an 11-item scale that evaluates various neurological deficits, with scores ranging from 0 (no deficits) to 42 (severe deficits). The mRS is a 6-point scale that assesses functional independence, with scores ranging from 0 (no symptoms) to 5 (severe disability requiring constant care). The MMSE is a 30-point questionnaire that tests cognitive domains such as orientation, attention, memory, language, and visuospatial skills, with lower scores indicating greater cognitive impairment.

Fatigue severity was assessed using the Fatigue Severity Scale (FSS), a widely used 9-item self-report questionnaire that measures the impact of fatigue on daily activities and quality of life. Each item is scored on a 7-point Likert scale, ranging from 1 (strongly disagree) to 7 (strongly agree). The total FSS score is calculated by averaging the scores of the nine items, with higher scores indicating more severe fatigue. A cut-off score of ≥ 4 was used to define clinically significant fatigue, consistent with previous studies (Lerdal et al., 2005; Ozyemisci-Taskiran et al., 2019). The FSS has demonstrated good internal consistency, reliability, and validity in various patient populations, including stroke survivors.

### MRI data acquisition

MRI data acquisition was performed within 3 days of admission using a 3.0 Tesla MRI scanner (Discovery MR750, GE Healthcare, Milwaukee, WI, USA). The scanning protocol included T1-weighted spin-echo sequences, resting-state BOLD fMRI, and diffusion tensor imaging (DTI) sequences. Resting-state fMRI data were acquired using an echo-planar imaging (EPI) sequence with the following parameters: TR = 2000 ms, TE = 30 ms, flip angle = 78°, FOV = 22.4 cm, matrix size = 64 × 64, slice thickness = 3.5 mm, spacing = 0.7 mm, number of slices = 36, and 240 volumes (8 min). During the rs-fMRI scan, participants were instructed to keep their eyes closed, stay awake, and not think of anything in particular. DTI data were obtained using a single-shot EPI sequence with the following parameters: TR = 5,665 ms, TE = minimum, flip angle = 90°, FOV = 22.4 cm, matrix size = 112 × 112, slice thickness = 3.5 mm, spacing = 0.7 mm, number of slices = 36, 64 diffusion gradient directions with b = 1,000 s/mm^2^, and 10 non-diffusion-weighted (b0) images. The acquisition time for the DTI sequence was 7 min and 5 s. To ensure high-quality MRI data, several quality control measures were implemented, including participant training to minimize head motion, use of foam padding and headphones, preprocessing of rs-fMRI data to correct for head motion, visual inspection of DTI data for artifacts, and calculation of relative signal-to-noise ratio (SNR) for each participant. Participants with excessive head motion, poor-quality DTI data, or low SNR (< 50) were excluded from the analysis.

### Data analysis

#### Rs-fMRI data analysis

The raw fMRI data were preprocessed using SPM12 (Wellcome Department of Cognitive Neurology, London, UK) in MATLAB (MathWorks, Natick, MA, USA). The first 10 volumes were discarded to allow for signal equilibration and participant adaptation. Preprocessing steps included slice-timing correction (with the middle slice as the timing reference), realignment for head motion correction, spatial normalization to the MNI standard space, and spatial smoothing with an 8-mm FWHM Gaussian kernel. For head motion control, participants with displacement exceeding 2.5 mm in any direction or angular motion exceeding 2.5 degrees were excluded from analysis. Six motion parameters (three translations and three rotations) were included as nuisance covariates in the first-level analysis. Additionally, we performed scrubbing to identify and exclude time points with excessive motion (framewise displacement > 0.5 mm).

Rs-fMRI data were preprocessed and analyzed using SPM12 (Wellcome Department of Cognitive Neurology, London, UK) in MATLAB (MathWorks, Natick, MA, USA). Following preprocessing, seed-based functional connectivity analysis was performed. The insula was selected as the seed region of interest (ROI) due to its involvement in fatigue processing. The insula was selected as the seed region of interest (ROI) based on several lines of evidence supporting its critical role in fatigue processing. First, the insula serves as a key hub of the salience network and plays a fundamental role in interoception—the awareness of internal bodily sensations including fatigue (Craig, 2009; Critchley et al., 2004). Second, previous neuroimaging studies have demonstrated altered insula function and connectivity in patients with fatigue across multiple clinical populations (Chien et al., 2015; Finke et al., 2015). Third, the insula has extensive anatomical and functional connections with multiple large-scale networks potentially relevant to PSF, including the default mode network, central executive network, and limbic system, making it an ideal candidate to investigate the complex network dynamics underlying PSF (Menon and Uddin, 2010). The mean time series from the insula ROI were extracted and correlated with time series from all other brain voxels using Pearson’s correlation coefficient. Correlation maps were then converted to z-maps using Fisher’s r-to-z transformation. A two-sample *t*-test was used to compare functional connectivity strength between the fatigue and non-fatigue groups, controlling for age, gender, and education as covariates. Statistical thresholds were set at *p* < 0.001 (uncorrected) at the voxel level and *p* < 0.05 (FWE-corrected) at the cluster level.

Statistical maps were overlaid on a standard MNI brain template using MRIcroGL. Significantly activated regions were labeled according to the AAL atlas. Coordinates, cluster sizes, and peak *t*-values of significant clusters were reported in a table.

A two-sample *t*-test was used to compare functional connectivity strength between the fatigue and non-fatigue groups, controlling for age, gender, and education as covariates. Statistical thresholds were set at *p* < 0.001 (uncorrected) at the voxel level and *p* < 0.05 (FWE-corrected) at the cluster level to control for multiple comparisons. For ROI-to-ROI analyses, false discovery rate (FDR) correction (*q* < 0.05) was applied across all tested connections.

### Handling of stroke lesions in connectivity analyses

To address the potential confounding effect of stroke lesions on functional connectivity analyses, we implemented several methodological strategies. First, we created individual lesion masks from diffusion-weighted images for each participant. These masks were co-registered to the standard space and used to identify and exclude lesioned tissue from our seed regions.

For participants with lesions affecting our seed regions (*n* = 7; 3 in PSF group, 4 in non-PSF group), we utilized the contralateral homologous region as the seed. Sensitivity analyses excluding these participants yielded consistent results with our primary analyses.

To control for potential effects of lesion characteristics, lesion volume was included as a covariate in all between-group statistical comparisons. Additionally, we performed a lesion overlap analysis comparing lesion distribution patterns between groups, which revealed no significant differences in lesion location patterns between PSF and non-PSF groups (*p* > 0.05, FDR-corrected), particularly in the insula and prefrontal regions central to our analysis.

### DTI-ALPS data analysis

DTI-ALPS data were obtained through a series of processing steps. First, the raw DTI data were preprocessed to ensure quality, including head motion correction, eddy current correction, and brain extraction. Next, the diffusion tensor model was fitted to the preprocessed DTI data to derive parameters such as fractional anisotropy (FA), mean diffusivity (MD), axial diffusivity (AD), and radial diffusivity (RD). The DTI-ALPS technique was then applied to perform diffusion tensor imaging along the perivascular spaces (PVS). This process involved extracting the white matter skeleton from the FA images, segmenting the skeleton into numerous segments corresponding to individual PVS, calculating colored-coded FA along the direction of each PVS segment, generating DTI-ALPS images based on the colored-coded FA, and computing the DTI-ALPS index to quantify the strength of water molecule diffusion along the PVS, which reflects the function of the glymphatic system.

DTI data were preprocessed and analyzed using the FMRIB Software Library (FSL) (Oxford University, Oxford, UK). After preprocessing, the DTI-ALPS index was calculated for each participant. An independent samples *t*-test was used to compare the DTI-ALPS index between the fatigue and non-fatigue groups. To investigate the association between the DTI-ALPS index and fatigue severity (FSS scores) in the fatigue group, a partial correlation analysis was performed, controlling for age, gender, and education. The DTI-ALPS technique was applied to assess water diffusion along perivascular spaces using several key parameters. For defining the direction of water diffusion, we utilized a combined approach of anatomical landmarks and probabilistic tractography. Specifically, major vascular structures were first identified using T1-weighted and T2-weighted images, and the principal diffusion direction was determined along these structures using a probability threshold of 0.8. ROI localization was performed using a semi-automated approach, where an initial automated segmentation of perivascular spaces was conducted using FSL’s FAST segmentation tool, followed by manual refinement by two experienced neuroradiologists who were blinded to participant group allocation. Inter-rater reliability was established with a kappa coefficient of 0.89. To minimize cerebrospinal fluid (CSF) contamination, we employed a rigorous CSF suppression procedure where voxels with free water fraction exceeding 0.5 (calculated using a bi-tensor model) were excluded from analysis. Additionally, we applied a 2 mm distance threshold from ventricular borders to further reduce CSF-related partial volume effects. The DTI-ALPS index was calculated as the mean value of directional diffusion along the defined perivascular pathways, with a fractional anisotropy threshold of 0.2 applied to exclude voxels with isotropic diffusion patterns.

An independent samples *t*-test was used to compare the DTI-ALPS index between the fatigue and non-fatigue groups. To investigate the association between the DTI-ALPS index and depressive symptoms in the fatigue group, a partial correlation analysis was performed, controlling for age, gender, and education. All statistical tests were two-tailed, and a *p*-value < 0.05 was considered statistically significant. Multiple comparisons were corrected using the FWE method for rs-fMRI data and the false discovery rate (FDR) method for DTI data.

### Statistical analyses

Clinical and demographic data were analyzed using SPSS 22.0 (IBM Corp., Armonk, NY) and *p* < 0.05 was considered statistically significant. Continuous variables were compared between the fatigue and non-fatigue groups using independent samples *t*-tests for normally distributed data or Mann–Whitney U tests for non-normally distributed data. Categorical variables were compared using chi-square tests or Fisher’s exact tests, as appropriate. To address the potential confounding effect of age differences between groups, we employed multiple strategies. First, age was included as a covariate in all functional connectivity analyses. Second, we conducted sensitivity analyses using age-matched subgroups (*n* = 35 per group) by excluding the five oldest participants from the PSF group and the fifteen youngest from the non-PSF group. Third, we examined within-group correlations between age and our primary imaging measures to assess whether age significantly influenced these metrics within each group.

Effect sizes for group comparisons were calculated using Cohen’s d, with values of 0.2, 0.5, and 0.8 representing small, medium, and large effects, respectively (Cohen, 1988). The 95% confidence intervals (CI) for Cohen’s d were calculated using the noncentral *t*-distribution method as recommended by Cumming (2014). These measures provide standardized estimates of the magnitude of observed differences and the precision of these estimates, enhancing the interpretability of our findings beyond statistical significance testing.

## Results

### Demographic and clinical characteristics

A total of 90 patients with first-ever ischemic stroke were enrolled in this study, including 40 patients with PSF and 50 without PSF (Table 1). The PSF group was significantly older than the non-fatigue group (62.70 ± 10.50 vs. 58.40 ± 9.20 years, *p* = 0.034). Given the significant age difference between groups, we performed sensitivity analyses with age-matched subgroups that confirmed the robustness of our primary findings. Within-group correlation analyses revealed no significant associations between age and our primary imaging measures (all *p* > 0.15), suggesting that age did not strongly influence the neuroimaging metrics within each group (Supplementary Tables S1, S2). There were no significant differences between groups in gender distribution (75.00% vs. 78.00% male, *p* = 0.773), BMI (24.90 ± 2.70 vs. 25.30 ± 2.80 kg/m^2^, *p* = 0.497), or education level (*p* = 0.892).

**Table 1:** Comparison of clinical characteristics between non-fatigue (*N* = 50) and fatigue (*N* = 40) groups.

Characteristics	Non-fatigue (*N* = 50)	Fatigue (*N* = 40)	*t*/z/χ^2^	*p*
Age (years), Mean ± SD	58.40 ± 9.20	62.70 ± 10.50	−2.156	0.034*
Gender, male (%)	39 (78.00)	30 (75.00)	0.083	0.773
BMI (kg/m^2^), Mean ± SD	25.30 ± 2.80	24.90 ± 2.70	0.682	0.497
Education (%)			Fisher	0.892
Illiterate	7 (14.00)	5 (12.50)		
Primary school	21 (42.00)	18 (45.00)		
Secondary and above	22 (44.00)	17 (42.50)		
Hypertension (%)	35 (70.00)	29 (72.50)	0.042	0.837
Diabetes (%)	12 (24.00)	14 (35.00)	1.342	0.247
Smoking history (%)	23 (46.00)	25 (62.50)	2.413	0.120
Drinking history (%)	20 (40.00)	18 (45.00)	0.236	0.627
TOAST classification (%)			Fisher	0.064
Large-artery atherosclerosis	18 (36.00)	20 (50.00)		
Small-vessel occlusion	28 (56.00)	16 (40.00)		
Cardioembolic	2 (4.00)	3 (7.50)		
Cryptogenic	2 (4.00)	1 (2.50)		
Stroke location (%)			Fisher	0.893
Cerebral hemisphere	32 (64.00)	24 (60.00)		
Cerebellum	5 (10.00)	3 (7.50)		
Brainstem	8 (16.00)	7 (17.50)		
Subcortical	4 (8.00)	6 (15.00)		
Mixed type	1 (2.00)	0 (0. 00)		
Infarct diameter (cm), Mean ± SD	1.45 ± 0.78	1.58 ± 0.92	−0.724	0.471
NIHSS score, Mean ± SD	4.82 ± 2.14	5.45 ± 2.36	−1.342	0.183
mRS score (%)			0.598	0.439
≤ 1	42 (84.00)	8(16.00)		
> 1	8 (16.00)	9 (22.50)		
MMSE score, Mean ± SD	27.40 ± 2.32	26.85 ± 2.64	1.056	0.294
FSS score, Mean ± SD	22.50 ± 8.40	48.30 ± 7.20	−15.324	<0.001***

BMI, Body Mass Index; TOAST, Trial of Org 10,172 in Acute Stroke Treatment; NIHSS, National Institutes of Health Stroke Scale; mRS, modified Rankin Scale; MMSE, Mini-Mental State Examination; FSS, Fatigue Severity Scale; SD, Standard Deviation. *p < 0.05, ***p < 0.001.

Vascular risk factors, including hypertension (72.50% vs. 70.00%, *p* = 0.837), diabetes (35.00% vs. 24.00%, *p* = 0.247), smoking history (62.50% vs. 46.00%, *p* = 0.120), and drinking history (45.00% vs. 40.00%, *p* = 0.627), were comparable between groups. According to the TOAST classification, there were no significant differences in stroke etiology between groups (*p* = 0.064), with small-vessel occlusion being the most common type in both groups (40.00% vs. 56.00%). Similarly, stroke location showed no significant differences between groups (*p* = 0.893), with cerebral hemisphere being the predominant location (60.00% vs. 64.00%).

The mean infarct diameter (1.58 ± 0.92 vs. 1.45 ± 0.78 cm, *p* = 0.471), NIHSS scores (5.45 ± 2.36 vs. 4.82 ± 2.14, *p* = 0.183), and MMSE scores (26.85 ± 2.64 vs. 27.40 ± 2.32, *p* = 0.294) were also compared between groups. As expected, the PSF group showed significantly higher FSS scores compared to the non-fatigue group (48.30 ± 7.20 vs. 22.50 ± 8.40, *p* < 0.001) (Table 1).

### Resting-state fMRI findings

**Figure 1:**
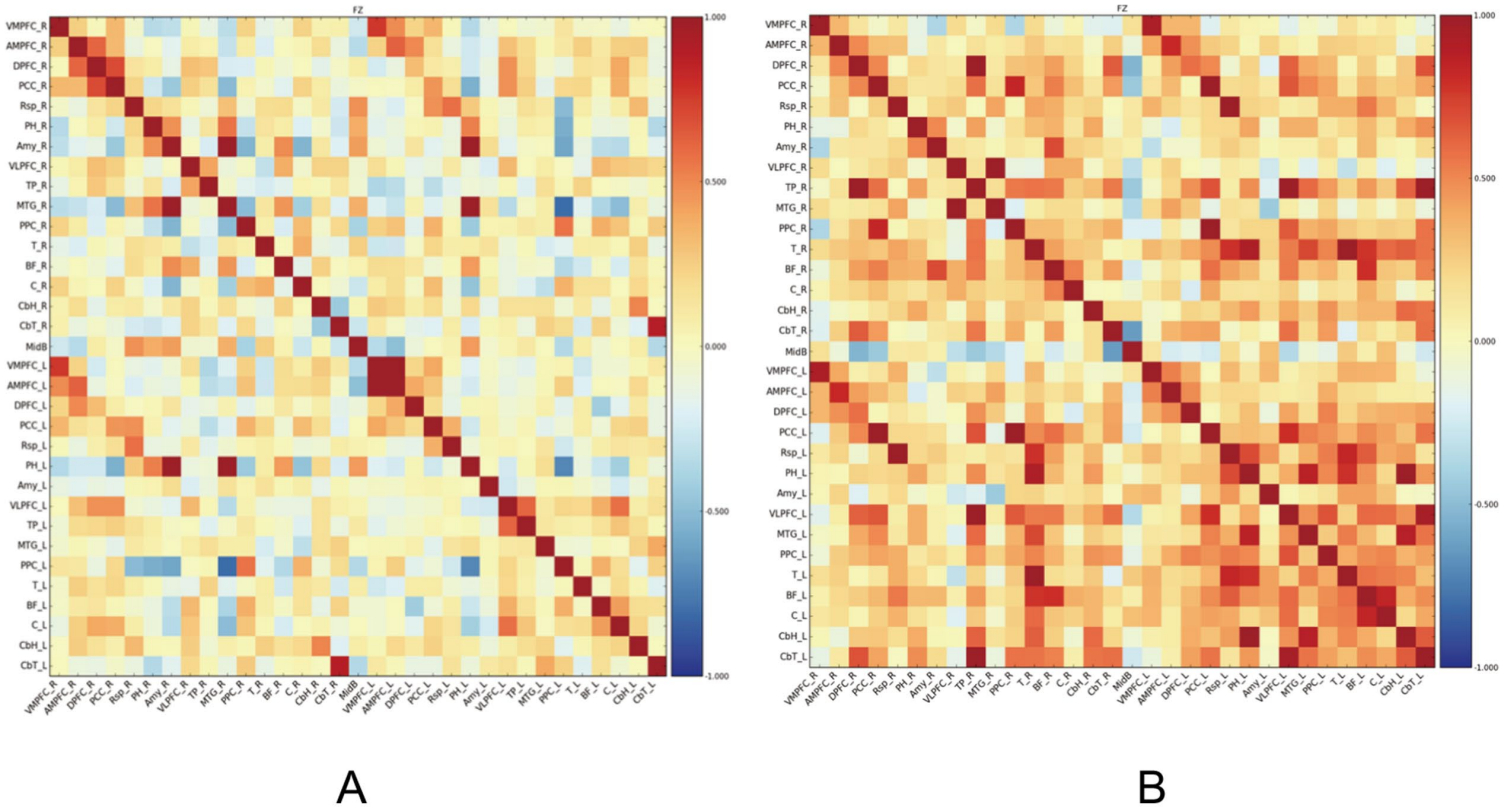
Functional connectivity matrices in stroke patients. **(A)** The left panel shows the functional connectivity matrix of the PSF group, demonstrating overall reduced connectivity. **(B)** The right panel shows the functional connectivity matrix of the non-PSF group, exhibiting generally stronger connectivity patterns. The color bar indicates correlation coefficient values ranging from −1 (dark blue) to 1 (dark red), where warmer colors represent stronger positive correlations and cooler colors represent negative correlations. The matrices are organized by brain regions according to anatomical parcellation, with the diagonal representing self-correlation of each region (*r* = 1.0). This visualization reveals distinct patterns of brain network organization between PSF and non-PSF groups, with PSF patients showing widespread reduction in functional connectivity.

**Figure 2:**
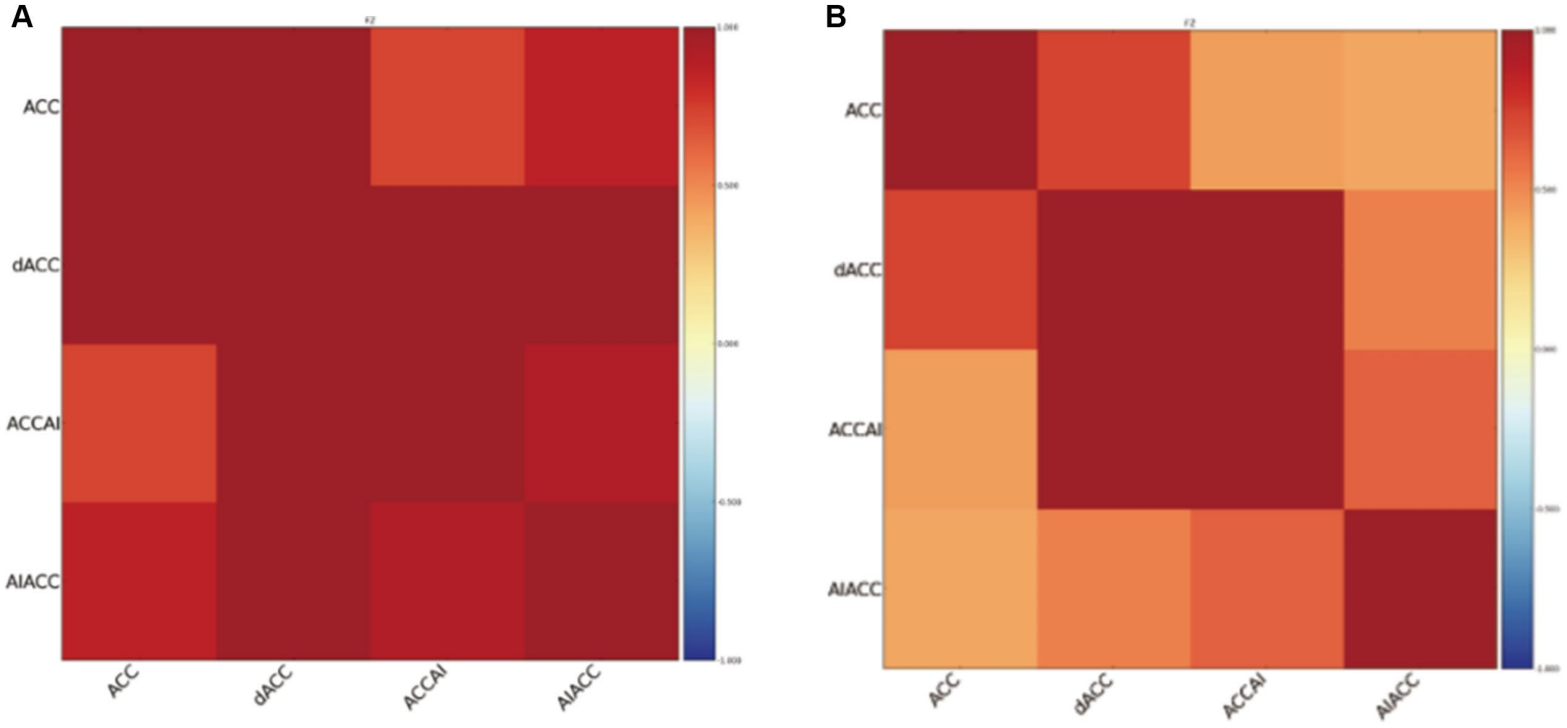
Functional connectivity matrices within the salience network in stroke patients. **(A)** The left panel shows the salience network connectivity matrix of the PSF group, demonstrating enhanced connectivity between key nodes. **(B)** The right panel shows the salience network connectivity matrix of the non-PSF group. The color bar represents correlation coefficient values from −1 (dark blue) to 1 (dark red). Brain regions include the anterior cingulate cortex (ACC), dorsal anterior cingulate cortex (dACC), anterior cingulate cortex/anterior insula (ACCAI), and anterior insula/anterior cingulate cortex (AIACC). The matrices reveal stronger functional connectivity within the salience network in PSF patients compared to non-PSF controls.

Resting-state functional connectivity analysis revealed significant differences between PSF and non-PSF groups. Compared with the non-PSF group, the PSF group showed significantly reduced global functional connectivity (0.28 ± 0.09 vs. 0.39 ± 0.11, *t* = −4.85, *p* < 0.01, Cohen’s d = −1.08, 95% CI [−1.45, −0.71]), as demonstrated in the whole-brain functional connectivity matrices (Figure 1). However, enhanced connectivity was observed in specific brain regions, particularly within the salience network (Figure 2).

Region-specific analysis showed that the functional connectivity strength between the insula and right inferior frontal operculum was significantly increased in the PSF group compared to the non-PSF group (0.42 ± 0.13 vs. 0.24 ± 0.10, *t* = 7.29, *p* < 0.001, Cohen’s d = 1.62, 95% CI [1.21, 2.03]). Additionally, the PSF group exhibited decreased functional connectivity between the precuneus and prefrontal cortex (0.19 ± 0.08 vs. 0.33 ± 0.11, *t* = −6.47, *p* < 0.001, Cohen’s d = −1.45, 95% CI [−1.84, −1.05]), while showing increased connectivity between the right insula and precuneus (0.37 ± 0.12 vs. 0.22 ± 0.09, *t* = 6.55, *p* < 0.01, Cohen’s d = 1.44, 95% CI [1.04, 1.83]) (Table 2). The lesion overlap analysis demonstrated that there were no significant differences in lesion distribution patterns between the PSF and non-PSF groups (*p* > 0.05, FDR-corrected). The functional connectivity alterations observed in the PSF group remained significant after controlling for lesion volume and location, suggesting that these connectivity patterns are specifically associated with post-stroke fatigue rather than direct consequences of structural damage.

**Table 2:** Comparison of functional connectivity strength in brain regions between fatigued and non-fatigued controls after stroke.

Brain area	Fatigue (*N* = 40)	Non-fatigue (*N* = 50)	*t*	*p*	Cohen’s d	95% CI
Integral functional connectivity	0.28 ± 0.09	0.39 ± 0.11	−4.85	<0.01**	−1.08	[−1.45, −0.71]
Insula-right inferior frontal operculum	0.42 ± 0.13	0.24 ± 0.10	7.29	<0.001***	1.62	[1.21, 2.03]
Precuneus-Prefrontal Cortex	0.19 ± 0.08	0.33 ± 0.11	−6.47	<0.001***	−1.45	[−1.84, −1.05]
Right Insula-Precuneus	0.37 ± 0.12	0.22 ± 0.09	6.55	<0.01**	1.44	[1.04, 1.83]

**p < 0.01, ***p < 0.001.

These findings suggest that PSF is characterized by a disruption of global brain functional integration, accompanied by specific regional connectivity alterations, particularly involving the salience network and default mode network components. The enhanced insula-related connectivity might reflect altered fatigue perception and emotional processing in PSF patients.

### DTI-ALPS findings

The DTI-ALPS indices were compared between the PSF and non-PSF groups to investigate the potential role of the glymphatic system in post-stroke fatigue. As shown in Table 3, the PSF group exhibited significantly lower DTI-ALPS indices compared to the non-PSF group in multiple brain regions, including the DTI-ALPS index (*t* = −5.23, *p* < 0.001, Cohen’s d = −1.22, 95% CI [−1.67, −0.77]), left hemisphere (*t* = −3.65, *p* < 0.01, Cohen’s d = −0.85, 95% CI [−1.28, −0.42]), right hemisphere (*t* = −3.34, *p* < 0.01, Cohen’s d = −0.78, 95% CI [−1.21, −0.35]), anterior region (*t* = −2.45, *p* < 0.05, Cohen’s d = −0.57, 95% CI [−0.99, −0.15]), and posterior region (*t* = −3.71, *p* < 0.01, Cohen’s d = −0.87, 95% CI [−1.29, −0.44]), after FDR correction (Table 3). These findings suggest that the PSF group had impaired glymphatic system function compared to the non-PSF group.

**Table 3:** Comparison of DTI-ALPS indices between PSF and non-PSF groups.

Variables	Fatigue (*N* = 40)	Non-fatigue (*N* = 50)	*t*	*p*	Cohen’s d	95% CI
DTI-ALPS index	1.15 ± 0.18	1.42 ± 0.25	−5.23	<0.001***	−1.22	[−1.67, −0.77]
Left hemisphere	1.12 ± 0.19	1.40 ± 0.21	−3.65	<0.01**	−0.85	[−1.28, −0.42]
Right hemisphere	1.10 ± 0.14	1.37 ± 0.19	−3.34	<0.01**	−0.78	[−1.21, −0.35]
Anterior region	1.14 ± 0.12	1.41 ± 0.22	−2.45	<0.05*	−0.57	[−0.99, −0.15]
Posterior region	1.11 ± 0.09	1.43 ± 0.17	−3.71	<0.01**	−0.87	[−1.29, −0.44]

Values are presented as mean ± standard deviation. FDR corrected. DTI-ALPS: diffusion tensor imaging along the perivascular space. *p < 0.05, **p < 0.01, ***p < 0.001.

**Figure 3:**
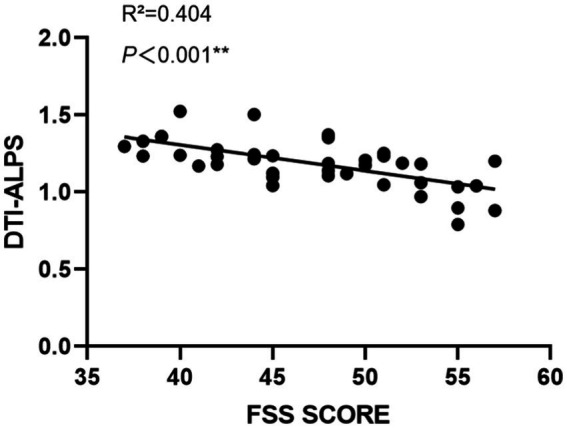
Correlation between DTI-ALPS indices and FSS scores in PSF patients. The scatter plot demonstrates a significant negative correlation between DTI-ALPS indices and FSS scores (R^2^ = 0.404, *p* < 0.001). Each dot represents an individual patient. The solid black line indicates the linear regression fit. The x-axis shows FSS scores ranging from 35 to 60, and the y-axis shows DTI-ALPS indices ranging from 0 to 2.0. This relationship suggests that higher fatigue severity is associated with lower glymphatic system function as measured by DTI-ALPS.

The relationship between the DTI-ALPS index and fatigue severity, as measured by the FSS score, was explored in the PSF group. As depicted in Figure 3, a significant negative correlation was observed between the DTI-ALPS index and FSS score (R^2^ = 0.404, *p* < 0.001), indicating that lower DTI-ALPS indices were associated with higher levels of fatigue severity in post-stroke patients. This finding highlights the potential link between glymphatic system dysfunction and the subjective experience of fatigue following stroke.

During the follow-up, a close relationship between white matter fiber bundle infarction and the persistent occurrence of PSF was discovered through the analysis of DTI data. Due to the sample size and the difficulty of follow-up, this article will not discuss this finding in depth. Further research in this area will be conducted in subsequent experiments.

The DTI-ALPS results provide evidence for impaired glymphatic system function in patients with post-stroke fatigue compared to those without fatigue. The negative correlation between the DTI-ALPS index and fatigue severity further supports the role of the glymphatic system in the pathophysiology of post-stroke fatigue. These findings offer new insights into the underlying mechanisms of fatigue after stroke and suggest that the glymphatic system may be a potential target for therapeutic interventions in the management of post-stroke fatigue.

## Discussion

The present study employed a multimodal neuroimaging approach, combining resting-state functional magnetic resonance imaging (rs-fMRI) and diffusion tensor imaging along the perivascular space (DTI-ALPS), to elucidate the neural correlates and potential mechanisms underlying post-stroke fatigue (PSF). The rs-fMRI results revealed altered functional connectivity patterns in the PSF group compared to the non-PSF group, particularly involving the insula, right inferior frontal gyrus (IFG) opercular part, precuneus, and prefrontal cortex. Specifically, the PSF group exhibited decreased functional connectivity between the precuneus and prefrontal cortex, and increased connectivity between the precuneus and right insula. These findings suggest that aberrant functional connectivity within the default mode network (DMN) may be closely associated with the pathogenesis of PSF, corroborating previous research (Ren et al., 2024). The DMN is implicated in self-referential processing, mind-wandering, and introspection (Raichle, 2015), and its dysfunction has been linked to various neuropsychiatric disorders, including depression and chronic fatigue syndrome (Buckner et al., 2008; Whitfield-Gabrieli and Ford, 2012). The altered DMN connectivity observed in this study may reflect maladaptive self-monitoring and increased attention toward internal fatigue sensations in PSF patients.

Our findings revealed an intriguing neurophysiological phenomenon: while PSF patients exhibited reduced global functional connectivity, they simultaneously showed enhanced connectivity between specific brain regions, particularly the insula and right inferior frontal gyrus (IFG) opercular part. This apparent paradox warrants careful interpretation. The reduced global connectivity likely represents the overall disruption of neural networks following stroke, consistent with previous studies demonstrating widespread alterations in network integration after cerebrovascular injury (Siegel et al., 2016). In contrast, the enhanced insula-rIFG connectivity represents a region-specific alteration that may have both pathological and compensatory implications.

From a pathological perspective, this enhanced connectivity within the salience network may reflect a maladaptive heightening of fatigue awareness. The insula plays a critical role in interoception—the perception of internal bodily sensations—while the right IFG is involved in attentional control and cognitive processing of salient stimuli (Menon, 2011). Hyperconnectivity between these regions could amplify the perception of fatigue signals, potentially explaining the disproportionate experience of fatigue relative to objective measures of physical exertion in PSF patients. Similar patterns of selective hyperconnectivity within generally disconnected networks have been observed in other neuropsychiatric conditions, such as chronic fatigue syndrome (Wortinger et al., 2016) and depression (Mulders et al., 2015).

Alternatively, or perhaps concurrently, this enhanced regional connectivity may represent a compensatory mechanism. In response to global network disruption, the brain may attempt to recruit additional neural resources by strengthening specific functional connections to maintain cognitive and emotional processing. Such compensatory hyperconnectivity has been documented in various neurological conditions and aging (Hillary and Grafman, 2017). The enhanced insula-rIFG connectivity could reflect the brain’s effort to regulate the subjective experience of fatigue by increasing communication between regions involved in interoception and cognitive control.

This complex interplay between global disconnection and regional hyperconnection underscores the multifaceted nature of PSF’s neural substrates and suggests that therapeutic approaches might need to target both the restoration of global network integration and the modulation of specific regional connectivity patterns.

Of particular interest is the enhanced connectivity between the insula and right IFG opercular part in the PSF group, as these regions constitute core nodes of the salience network (SN). The SN is responsible for detecting and orienting attention toward salient stimuli, and its dysregulation has been implicated in a range of neuropsychiatric conditions, including chronic fatigue syndrome (Menon, 2011; Wortinger et al., 2016). The increased connectivity within the SN observed in this study may indicate heightened sensitivity to fatigue-related stimuli and an attentional bias toward fatigue sensations in PSF patients. This interpretation is consistent with the cognitive-behavioral model of chronic fatigue syndrome, which posits that excessive attention to fatigue symptoms and maladaptive illness beliefs perpetuate the experience of fatigue (Knoop et al., 2008; Vercoulen et al., 1998). The interplay between the DMN and SN in the context of PSF warrants further investigation, as the dynamic interaction between these networks may underlie the cognitive and affective aspects of fatigue (Menon, 2011).

The DTI-ALPS findings complement the rs-fMRI results by revealing significantly lower DTI-ALPS indices in the PSF group compared to the non-PSF group, indicative of impaired glymphatic system function. The glymphatic system, a brain-wide network of perivascular channels, facilitates the clearance of neurotoxic waste products and inflammatory mediators from the brain parenchyma (Iliff et al., 2012; Nedergaard, 2013). In the context of PSF, impaired glymphatic function may lead to the accumulation of neurotoxic substances and inflammatory mediators, which could exacerbate neuronal dysfunction and contribute to the development and maintenance of fatigue symptoms. Interestingly, a recent study by Xie et al. (2013) demonstrated that sleep is a critical regulator of glymphatic function. Using *in vivo* two-photon imaging in mice, they found that natural sleep or anesthesia was associated with a 60% increase in the interstitial space, resulting in a striking increase in convective exchange of cerebrospinal fluid with interstitial fluid compared to the awake state. This sleep-induced enhancement of glymphatic influx and efflux was accompanied by accelerated clearance of *β*-amyloid, a neurotoxic waste product, from the brain. Given the high prevalence of sleep disturbances in stroke survivors and the well-established link between sleep and fatigue, the findings of impaired glymphatic function in the PSF group, coupled with the sleep-dependent regulation of glymphatic clearance, provide a compelling mechanistic framework for understanding the complex interplay between sleep, neuroinflammation, and fatigue in the context of stroke.

The accumulation of neurotoxic waste products and inflammatory mediators due to impaired glymphatic clearance during sleep may exacerbate neuronal dysfunction and microglial activation, leading to the development and perpetuation of fatigue symptoms. This hypothesis aligns with the cognitive-behavioral model of fatigue, which posits that aberrant inflammatory processes and maladaptive cognitive responses to fatigue sensations interact to maintain the fatigue state (Knoop et al., 2008; Vercoulen et al., 1998). The DTI-ALPS findings, in conjunction with the rs-fMRI results and the growing literature on the glymphatic system, suggest that targeting the glymphatic pathway and its regulators, such as sleep, may offer novel therapeutic opportunities for alleviating PSF.

The multimodal neuroimaging approach employed in this study offers several advantages over single-modality investigations. By combining rs-fMRI and DTI-ALPS, we were able to assess both functional brain networks and glymphatic system integrity, providing a more comprehensive understanding of the neural mechanisms underlying PSF. The parallel analysis of these complementary techniques allowed for the identification of convergent abnormalities in the PSF group, enhancing the robustness and validity of our findings. Moreover, the use of DTI-ALPS represents a technical innovation, as it enables the non-invasive assessment of glymphatic function *in vivo*, which was previously only possible through invasive methods (Taoka and Naganawa, 2020). This novel approach opens new avenues for investigating the role of the glymphatic system in various neurological and psychiatric disorders, and may facilitate the development of targeted therapeutic interventions.

The current findings have important implications for the diagnosis and treatment of PSF. The altered functional connectivity patterns and impaired glymphatic function observed in the PSF group could potentially serve as neuroimaging biomarkers for the early identification of patients at risk of developing PSF. This is particularly relevant given the high prevalence of PSF and its significant impact on quality of life and rehabilitation outcomes in stroke survivors (Cumming et al., 2016). The identification of reliable biomarkers could facilitate the implementation of preventive strategies and personalized treatment approaches for PSF. Moreover, the findings suggest that targeting the DMN, SN, and glymphatic system may be promising therapeutic strategies for alleviating fatigue symptoms in stroke survivors. For example, non-invasive brain stimulation techniques, such as transcranial magnetic stimulation (TMS) or transcranial direct current stimulation (tDCS), could be used to modulate the activity of key regions within the DMN and SN. Additionally, interventions aimed at promoting glymphatic function, such as exercise or pharmacological agents (e.g., aquaporin-4 agonists), may help to reduce fatigue by enhancing the clearance of neurotoxic substances (Benveniste et al., 2017; von Holstein-Rathlou et al., 2018). The development of novel therapeutic strategies targeting the neural mechanisms underlying PSF is an important goal for future research, as current treatment options for PSF are limited and often inadequate (Wu et al., 2014).

Despite the novel findings, this study has several limitations that should be addressed in future research. First, the sample size was relatively small, which may limit the generalizability of the results. Future studies should employ larger, more diverse samples to validate the current findings and explore potential moderators of neural alterations in PSF, such as age, sex, and stroke characteristics. Second, the cross-sectional design precludes causal inferences about the relationship between functional connectivity, glymphatic function, and PSF. Longitudinal studies are needed to investigate the temporal dynamics of neural changes in PSF and to determine whether the observed alterations are a cause or consequence of fatigue. Third, while the multimodal approach used in this study provides a more comprehensive understanding of the neural mechanisms underlying PSF, the inclusion of additional neuroimaging modalities, such as positron emission tomography (PET) or magnetic resonance spectroscopy (MRS), could offer further insights into the metabolic and neurochemical alterations associated with PSF. Future studies should consider combining multiple neuroimaging techniques to gain a more holistic view of the neural correlates of PSF. Last but not least, we acknowledge several technical limitations of the DTI-ALPS methodology that may impact the interpretation of our findings. First, despite our efforts to minimize partial volume effects through CSF suppression techniques, some contamination from non-perivascular tissue may persist, particularly in regions with complex microstructural organization. Second, the DTI-ALPS approach relies on diffusion tensor metrics that cannot fully resolve crossing fibers or complex diffusion geometries, potentially leading to underestimation of water diffusion along perivascular spaces in regions with crossing fiber architectures. Third, the technique’s sensitivity to acquisition parameters (including b-value selection, number of diffusion directions, and spatial resolution) introduces variability that may affect reproducibility. Fourth, while DTI-ALPS provides indirect measurement of glymphatic function, it cannot directly visualize the dynamic clearance process that occurs in real-time, limiting our ability to capture temporal dynamics of glymphatic function. Finally, despite promising clinical applications, the DTI-ALPS methodology still lacks standardized computational approaches and comprehensive validation against invasive glymphatic assessment techniques, necessitating cautious interpretation of our findings. Future studies employing complementary techniques such as intrathecal contrast-enhanced MRI or dynamic contrast-enhanced MRI alongside DTI-ALPS would strengthen the validity of these measurements.

In conclusion, this multimodal neuroimaging study provides novel evidence for the involvement of functional brain networks and the glymphatic system in the pathophysiology of post-stroke fatigue. The findings highlight the potential of rs-fMRI and DTI-ALPS as valuable tools for understanding the neural mechanisms underlying PSF and developing targeted diagnostic and therapeutic strategies. The altered functional connectivity patterns within the DMN and SN, coupled with impaired glymphatic function, may represent key neural substrates of fatigue in stroke survivors. These results underscore the importance of considering both functional and structural brain alterations in the study of PSF, and emphasize the need for multimodal approaches in the investigation of complex neuropsychiatric symptoms. As neuroimaging techniques continue to advance, the integration of multiple modalities will become increasingly crucial for unraveling the intricate neural underpinnings of PSF and other neuropsychiatric comorbidities in stroke survivors. Future research should aim to replicate and extend the current findings, explore the clinical applications of multimodal neuroimaging in the management of PSF, and investigate the efficacy of novel therapeutic interventions targeting the identified neural mechanisms. Ultimately, a better understanding of the neural basis of PSF may lead to the development of more effective diagnostic tools and personalized treatment strategies, improving the quality of life and rehabilitation outcomes for stroke survivors worldwide.

## Conclusion

This multimodal neuroimaging investigation revealed significant alterations in brain network connectivity and glymphatic function in post-stroke fatigue patients. The PSF group exhibited decreased global functional connectivity but enhanced connectivity between the insula and right inferior frontal gyrus (IFG) opercular part, suggesting salience network dysfunction. Furthermore, reduced functional connectivity between the precuneus and prefrontal cortex, coupled with increased connectivity between the precuneus and right insula, indicated default mode network aberrations. Notably, DTI-ALPS analysis demonstrated impaired glymphatic system function in PSF patients, potentially contributing to fatigue pathophysiology through compromised waste clearance.

These findings carry substantial clinical implications. The identified patterns of altered functional connectivity and reduced DTI-ALPS indices could serve as potential biomarkers for PSF, facilitating early detection and intervention. The combination of rs-fMRI and DTI-ALPS provides a comprehensive framework for understanding PSF mechanisms, suggesting multiple therapeutic targets. Interventions targeting network connectivity modulation and glymphatic system enhancement may offer novel treatment strategies for PSF patients.

Future research should focus on longitudinal studies to elucidate the temporal evolution of these neural alterations and their relationship with fatigue symptoms. The incorporation of additional imaging modalities and larger sample sizes will further validate these findings and potentially uncover new therapeutic targets. This work establishes a foundation for developing personalized treatment approaches and improving rehabilitation outcomes in stroke survivors with fatigue.

## Data Availability

The original contributions presented in the study are included in the article/Supplementary material, further inquiries can be directed to the corresponding authors.
