# Supplementary material for: Multimodal neuroimaging investigation of post-stroke fatigue in middle-aged and older adults: combining resting-state fMRI and DTI-ALPS analysis
Source: Front Aging Neurosci. 2025 May 13;17:1583655. doi: 10.3389/fnagi.2025.1583655 (PMC12106435; doi:10.3389/fnagi.2025.1583655)
Supplement: Supplementary file 1 [file Table_1.docx]

Table S1. Comparison of functional connectivity strength in brain regions between age-matched PSF and non-PSF subgroups (n=35 per group)

| **Brain area** | **Fatigue**  **(N = 35)** | **Non-fatigue (N = 35)** | **t** | **p** |
| --- | --- | --- | --- | --- |
| Integral Functional Connectivity | 0.30 ± 0.09 | 0.36 ± 0.08 | -3.74 | 0.002** |
| Insula- right inferior frontal operculum | 0.39 ± 0.14 | 0.26 ± 0.16 | 5.85 | <0.001*** |
| Precuneus-Prefrontal Cortex | 0.21 ± 0.09 | 0.30 ± 0.11 | -4.92 | <0.001*** |
| Right Insula- Precuneus | 0.34 ± 0.13 | 0.25 ± 0.10 | 4.64 | 0.003** |

**P < 0.01, ***P < 0.001

Table S2. Comparison of DTI-ALPS indices between age-matched PSF and non-PSF subgroups (n=35 per group)

| **Variables** | **Fatigue**  **(N = 35)** | **Non-fatigue (N = 35)** | **t** | **p** |
| --- | --- | --- | --- | --- |
| DTI-ALPS index | 1.11 ± 0.16 | 1.37 ± 0.25 | -4.91 | <0.001*** |
| Left hemisphere | 1.08 ± 0.17 | 1.38 ± 0.20 | -3.58 | <0.01** |
| Right hemisphere | 1.13 ± 0.15 | 1.32 ± 0.23 | -3.43 | <0.01** |
| Anterior region | 1.14 ± 0.18 | 1.36 ± 0.21 | -2.62 | 0.021* |
| Posterior region | 1.07 ± 0.11 | 1.39 ± 0.24 | -3.84 | <0.01** |

Values are presented as mean ± standard deviation. FDR corrected. DTI-ALPS: diffusion tensor imaging along the perivascular space. *P < 0.05, **P < 0.01, ***P < 0.001
